# Editorial: Phage therapy in infectious diseases of animals and humans

**DOI:** 10.3389/fcimb.2023.1334273

**Published:** 2023-12-07

**Authors:** Alicja Węgrzyn

**Affiliations:** Phage Therapy Center, University Center for Applied and Interdisciplinary Research, University of Gdansk, Gdansk, Poland

**Keywords:** bacterial pathogens, bacteriophage therapy, avian diseases, human diseases, food

Bacteriophage therapy, or phage therapy, is the method of treatment of bacterial infections using bacteriophages, or phages, i.e. viruses which multiply in bacterial cells. It is considered as a potential therapy of human and animal diseases caused by pathogenic bacteria (Baral, 2023; Strathdee et al., 2023). Especially, this kind of therapy should be taken into consideration in the era of the antibiotic resistance crisis which is caused by appearance of bacterial strains resistant to many, if not all, currently available antibiotics (Tang et al., 2023). Nevertheless, despite many years of studies on phage therapy, this method remains largely at the level of laboratory studies, or experimental therapy at best. There are still controversies on its efficacy and safety, particularly when used at the large scale in human and veterinary medicine (Ali et al., 2023). In fact, despite some successes in the use of experimental phage therapy, the efficacy and safety issues are still unclear, mainly due to only limited experimental approaches reported in individual publications, particularly the use of a low number of strains and measurement of only a few parameters of efficacy and safety profile (Podlacha et al., 2021; Singh et al., 2023). Therefore, further studies should provide data which will add important input to our knowledge on effects of bacteriophages administered to animals and humans infected with virulent bacterial strains. We are still awaiting more studies which should be complex and focused on global analysis of interactions between bacteria and phages in animals and humans, and also on analysis of putative interactions between bacteriophages and organisms of investigated animal and human organisms infected with pathogenic bacteria. Cellular responses to treatment with bacteriophages should also be studied at molecular levels. This Research Topic has been devoted to works focused on development of phage therapy in different aspects, including human infections, animal infections, and food protection. A collection of four articles is finally included in this Research Topic. These papers describe original works in the areas of combating avian infections with *Salmonella enterica* and *Escherichia coli* strains, emergence of *Salmonella* isolates with reduced susceptibility to bacteriophages in the light of food protection and therapy of chickens, and eradication of *Staphylococcus capitis* from neonatal incubators.

It appears that that introduction of phage therapy to the practice should be easier and quicker in veterinary medicine than in the treatment of human diseases. In the first article from this series, efficacy and safety of phage therapy was compared with those of the use of antibiotics in the model of chicken infection with *S. enterica* serovar Typhimurium (Kosznik-Kwaśnicka et al.). A cocktail of two bacteriophages and two antibiotics (enrofloxacin and colistin) were investigated. Efficiencies of both therapies in eradicating *S.* Typhimurium from chicken gastrointestinal tract were found high and similar, especially when the therapeutic agents were applied shortly (up to 24 h) after infection. The presence of orally-administered bacteriophages in various organs of chickens could be detected, indicating that these viruses can be spread throughout the organism, though with a limited efficiency. Importantly, the researches were unable to detect an appearance of the bacterial resistance to phages and antibiotics during the whole experiment. When safety of the treatment was considered, it was confirmed that treatment with antibiotics caused significant changes in the chicken gut microbiota composition, and such changes remained for at least a month. In contrast, changes in the microbiome caused by application of bacteriophages were transient, and the composition of gut microbiota normalized within a few weeks. That study indicated that phage therapy can be as effective as antibiotic therapy in combating *S.* Typhimurium-caused infections in chickens, while the former treatment is more safe as causing less pronounced and shorter changes in the gut microbiome.

The problem of appearance of bacteriophage-resistance in *S*. Typhimurium was addressed in another work published in this Research Topic (López-Pérez et al.). The authors tested the efficiency of this phenomenon under various conditions, including (i) cultivation under laboratory conditions, (ii) biocontrol of cooked ham slices (a model for the food protection procedures), and (iii) oral phage therapy in broilers. The appearance of the resistance to bacteriophages was the quickest and the most effective in bacterial laboratory cultures. This resistance was mostly due to selection of mutants, especially in *rfc* and *rfaJ* genes, coding for proteins required for lipopolysaccharide synthesis. Bacteriophage-resistant mutants were significantly less abundant in the experiments with cooked ham slices. On the other hand, resistance to bacteriophages was rare in the phage therapy of broilers, and this phenomenon resulted from the lateral gene transfer of large plasmids from the IncI1 group, rather than from selection of *S*. Typhimurium mutants in specific genes. These differences in the type and frequency of appearance of bacteriophage resistance under different conditions is especially important in the light of development of the phage therapy in poultry. Moreover, the authors of the above presented article concluded that the relatively low efficiency of appearance of bacteriophage-resistant bacteria should not significantly affect the development of *S.* Typhimurium biocontrol and oral phage therapy.

Development of effective phage therapy requires isolation and characterization of a large set of bacteriophages, capable of infecting different bacterial species and strains. This type of work is exemplified by the article by Yao et al. who isolated and described properties of bacteriophage PEC9 which effectively infects *E. coli* strains pathogenic to birds.

The fourth article describes studies on the use of phage cocktails in combating bacterial biofilms formed in neonatal incubators by *S. capitis* (Chavignon et al.). The use of bacteriophages resulted in decreasing the number of bacterial cells in both dry spots and biofilms. Importantly, no appearance of bacterial resistance to phages could be detected during these experiments.

In summary, results described in this Research Topic confirmed a proof-of-concept for the use of bacteriophages in treatment of poultry infected with pathogenic strains of *S. enterica* and *E. coli*, and the use of phage cocktails in eradicating biofilms formed by *S. capitis*. Readers are encouraged to get acquainted with details of the works summarized above, as they provided important information, useful for further development of phage therapy procedures.

## Author contributions

AW: Writing – original draft, Writing – review & editing.
